# Association Between Sleep Quality and Oxidative Stress in Postoperative Cardiovascular Patients—A Pilot Study

**DOI:** 10.1111/jsr.70383

**Published:** 2026-06-01

**Authors:** K. Krawczyk, D. Riemann, E. Schleicher, A. Günther, A. Ayekoi, M. Czerny, J. S. Pooth, T. Böhmerle, B. Feige, J. Sahlmann, F. Beyersdorf

**Affiliations:** ^1^ Department of Cardiovascular Surgery University Hospital Freiburg Freiburg im Breisgau Germany; ^2^ Department of Radiology University Hospital Freiburg Freiburg im Breisgau Germany; ^3^ Faculty of Medicine Albert‐Ludwigs‐University Freiburg Freiburg im Breisgau Germany; ^4^ Department of Psychiatry and Psychotherapy University Hospital Freiburg Freiburg im Breisgau Germany; ^5^ Institute of Physical Chemistry, Albert‐Ludwigs‐University Freiburg Freiburg im Breisgau Germany; ^6^ Department of Emergency Medicine University Hospital Freiburg Freiburg im Breisgau Germany; ^7^ Institute for Medical Biometry and Statistics, Medical Center—University of Freiburg Freiburg im Breisgau Germany

**Keywords:** perioperative period, reactive oxygen species, sleep pattern

## Abstract

Oxidative stress contributes to numerous physiological and pathological processes, and sleep behaviour as well as circadian rhythms may influence reactive oxygen species (ROS) concentrations in humans. This prospective pilot study quantified postoperative venous ROS concentrations in patients undergoing cardiovascular surgery and explored associations with subjective and objective sleep parameters, circadian patterns and patient characteristics. Fifteen patients undergoing cardiovascular surgery at the University Medical Center Freiburg were monitored during the first 48 postoperative hours. Serial venous blood samples were analysed for ROS concentrations using electron spin resonance. Sleep was assessed using validated questionnaires, including the Insomnia Severity Index, Pittsburgh Sleep Quality Index and Schlaffragebogen‐A (SF‐A), as well as actigraphy. Additional clinical data were recorded. Venous ROS concentrations showed substantial inter‐individual variability but no significant circadian pattern. Mean postoperative ROS concentrations were not significantly associated with ISI scores, PSQI global score, SF‐A parameters or objective actigraphic sleep measures. Among PSQI subdomains, however, higher ROS concentrations were significantly associated with reduced sleep efficiency (*r* = −0.74, *p* = 0.002) and longer nocturnal wake time (*r* = 0.62, *p* = 0.01), while shorter sleep duration showed a trend‐level association (*r* = −0.49, *p* = 0.07). These findings demonstrate the feasibility of electron spin resonance‐based venous ROS quantification in postoperative cardiac surgery patients. Specific subjective sleep parameters, particularly reduced sleep efficiency and increased nocturnal wake time, may be associated with higher postoperative ROS concentrations. Larger studies are warranted to confirm these exploratory findings.

## Graphical abstract

In 15 postoperative cardiac surgery patients, venous reactive oxygen species (ROS) measured by electron spin resonance (ESR) over 48 h showed no consistent circadian pattern. Higher ROS was associated with lower subjective sleep efficiency and more nocturnal awakenings, but not with other sleep measures. ESR‐based ROS monitoring is feasible and may inform postoperative recovery.

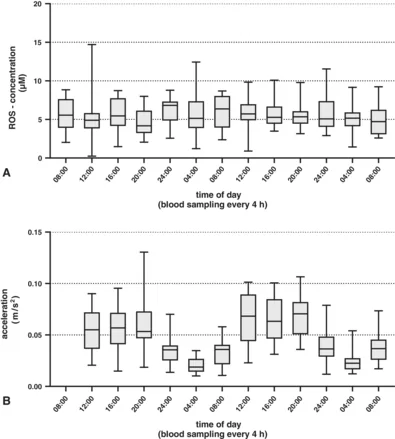

## 1 Introduction

Oxidative stress refers to an imbalance between the production of mainly reactive oxygen species (ROS) and the body's antioxidant defence capacity (Sies et al. 2022; Sies et al. 2024). While low levels of ROS are essential for processes such as cell signalling and immune responses, elevated concentrations can damage proteins, lipids and nucleic acids (Weidinger and Kozlov 2015). Persistent oxidative stress is implicated in the pathogenesis of cardiovascular, neurodegenerative and metabolic diseases (Cheng et al. 2022).

Experimental studies suggest that sleep and circadian regulation influence redox homeostasis: Sleep deprivation alters antioxidant gene expression (Anafi et al. 2013) and promotes ROS accumulation in the gut (Vaccaro et al. 2020). In contrast, adequate sleep protects neuronal membranes against oxidative damage (Rorsman et al. 2025; Hill et al. 2018). In humans, observational studies from shift workers indicate that chronic circadian misalignment and insufficient sleep are associated with increased levels of oxidative stress and reduced antioxidant capacity (Teixeira et al. 2019).

Postoperative patients, particularly after cardiovascular surgery, represent a high‐risk group for oxidative stress due to anaesthesia, surgical trauma, cardiopulmonary bypass, aortic crossclamping, systemic inflammation (Sugita and Fujiu 2018) and transfusions (Beyersdorf 2024). These same factors may also disrupt sleep and circadian rhythms. Moreover, the intensive care environment—with constant noise, artificial light exposure and frequent nocturnal disturbances—can further hinder recovery by a combination of sleep disturbances, circadian disruption and oxidative stress (Beyersdorf 2024).

Despite evidence from experimental models showing that (a) sleep deprivation induces oxidative stress (Anafi et al. 2013), and may even cause death through ROS accumulation in the gut (Vaccaro et al. 2020), and (b) adequate sleep protects neuronal membranes from oxidative damage (Rorsman et al. 2025; Hill et al. 2018), human data linking postoperative oxidative stress with sleep quality and circadian regulation remain scarce. Given the limited available evidence, the present study was designed as an exploratory investigation aiming (1) to quantify postoperative venous ROS concentrations using electron spin resonance (ESR), (2) to assess associations with subjective sleep, (3) to examine relationships with clinical variables, and (4) to investigate potential circadian patterns in postoperative ROS profiles.

## 2 Methods

### 2.1 Study Design and Ethics

This prospective pilot study was conducted at the Department of Cardiovascular Surgery, University Medical Center Freiburg, Germany. The study was approved by the Ethics Committee of the Albert‐Ludwigs‐University Freiburg (reference number 21‐1625) and registered in the German Clinical Trials Register (DRKS00021046). All patients provided written informed consent.

### 2.2 Participants

Between January and July 2022, 20 patients scheduled for elective cardiovascular surgery were enrolled. Inclusion criteria were age ≥ 18 years, expected postoperative hospital stay ≥ 72 h, and central venous catheter (CVC) in place for at least 48 h. Exclusion criteria included active infection, malignancy, BMI < 20 kg/m^2^, pregnancy, emergency procedures or inability to consent. Of the 20 enrolled patients, 15 completed the full study protocol. Five participants did not complete all planned measurements. Reasons for non‐completion included early removal of the central venous catheter (*n* = 3; after 1, 2 and 3 measurements, respectively) and participant burden related to the study procedures (*n* = 2; after 1 and 2 measurements, respectively).

### 2.3 Sampling and Sleep Assessment

Thirteen postoperative venous blood samples were collected via CVC every 4 h for 48 h. Patients simultaneously wore actigraphy sensors to monitor rest‐activity cycles (objective sleep assessment). Subjective sleep was assessed preoperatively and on both postoperative nights using validated questionnaires (Insomnia Severity Index (ISI) (Bastien 2001), Pittsburgh Sleep Quality Index (PSQI) (Buysse et al. 1989), Sleep Questionnaire (SF‐A) (Görtelmeyer 1981)).

### 2.4 Objective Sleep Monitoring

Patients wore wrist‐mounted actigraphy sensors (Move 4, movisens GmbH) continuously for 48 h. Devices recorded activity, sleep duration, sleep efficiency and restlessness. Data were processed using manufacturer software in collaboration with the Department of Psychiatry and Psychotherapy. As actigraphy has limited validity in distinguishing sleep from quiet wakefulness, especially in the absence of movement, it was used as a surrogate measure of sleep. Thus, undetected periods of quiet wakefulness may have led to an overestimation of the difference between daytime and nighttime activity. Activity‐related measures were derived using the device‐specific algorithm based on acceleration and barometric signals. Energy expenditure was estimated indirectly using an activity‐class‐based model that incorporates MovementAcceleration, altitude change and individual characteristics (age, sex, weight and height), and should therefore be regarded as an inferred rather than directly measured parameter (Härtel et al. 2011; Anastasopoulou et al. 2014; Armbruster et al. 2018).

### 2.5 Blood Sample Processing and Electron Paramagnetic Resonance (EPR) Sample Preparation

A degassed NaCl solution (0.9%, w/v) containing 25 μM deferoxamine mesylate (Sigma Aldrich, USA) and 5 μM sodium diethyldithiocarbamate (Roth, Karlsruhe, Germany) was prepared. 1.4 mM of N‐(1‐hydroxy‐2,2,6,6‐tetramethylpiperidin‐4‐yl)‐2‐methylpropanamide hydrochloride (TMTH‐HCl, Enzo Life Sciences, Farmingdale, USA) was added at 0°C under argon atmosphere. Thereafter, 100 μL of this solution were transferred into citrate monovettes (S‐Monovette 1.4 mL, Sarstedt, Germany) under argon atmosphere and immediately flash‐frozen in liquid nitrogen. Prior to usage, the prepared monovettes were thawed, and blood was transferred using a transfer tube into the monovettes yielding a total volume of 1.4 mL and a final TMTH concentration of 100 μM. After mixing the blood/TMTH solution for 5 s, the monovettes were flash‐frozen in liquid nitrogen. The monovettes were thawed at room temperature, then incubated at 37°C for 10 min and stored on ice until measurement. 30 μL of the blood‐TMTH solution was transferred into glass capillaries (1 mm inner diameter) using a Hamilton syringe directly before EPR measurement.

### 2.6 EPR Spectroscopy

All EPR measurements were conducted on an X‐band EMX‐nano spectrometer (Bruker Biospin, Germany) at room temperature using the following experimental parameters: microwave power = 6.31 mW, conversion time = 30 ms, receiver gain = 50 dB, modulation frequency = 100 kHz, and modulation amplitude = 3 G. An EPR spectrum spanning 300 G was recorded for each sample (central field = 3441.4 G, 1000 points, 4 scans). All spectra were baseline corrected by subtracting a first‐order polynomial. Subsequently, the spin concentration was calculated by the Xenon spin counting software (Bruker Biospin, Germany) using the following parameters: Sample diameter = 1 mm, sample centre = 125 mm, sample length = 25 mm and electron spin = 1/2 (The conversion of the double integral of the EPR signal to a concentration was performed using a calibration curve with 4‐oxo TEMPO) (Babic and Peyrot 2019).

### 2.7 Clinical Data and Statistical Analysis

Additional variables included surgical type, medication use and perioperative complications. Statistical analysis was performed using GraphPad Prism 9. Normality was assessed using the D'Agostino–Pearson omnibus test. For correlation analyses, mean ROS concentrations per patient were used to account for repeated measurements.

Mean postoperative ROS concentration was treated as the dependent variable, and subjective sleep parameters were analysed as independent variables. Correlations between mean postoperative ROS concentrations and subjective sleep parameters were analysed using Pearson or Spearman correlation coefficients, depending on data distribution and measurement level. Pearson correlation was applied to continuous variables meeting assumptions of normality and linearity, whereas Spearman rank correlation was used for ordinal or non‐normally distributed variables. Paired comparisons between pre‐hospitalisation (PSQI) and in‐hospital sleep parameters (SF‐A) were performed for conceptually comparable variables using the Wilcoxon signed‐rank test. Circadian variation in ROS was explored using 24‐h cosinor analysis at the group and individual level. All tests were two‐tailed with a significance level of *p* < 0.05. Given the small sample size, analyses were considered exploratory.

## 3 Results

### 3.1 Patient Characteristics

The study included 15 patients (9 male, 6 female), mean age 63.9 ± 10.9 years, mean body mass index (BMI) 29.1 ± 3.5 kg/m^2^. Most underwent coronary artery bypass grafting (CABG, *n* = 8), with others receiving aortic valve replacement (*n* = 2) or other procedures (*n* = 5), including left ventricular assist device (LVAD), atrial septal defect (ASD) repair, Leriche syndrome, aortic arch replacement and cardiac resynchronisation therapy (CRT) system implantation.

### 3.2 Subjective Sleep Outcomes

The average ISI score was 10.0 ± 6.1, consistent with subthreshold insomnia; only one third (5/15) reported no clinically significant insomnia. PSQI scores indicated impaired baseline sleep in 53% (8/15) (mean score 7.2 ± 2.3), so‐called ‘poor sleepers’. Paired comparisons between pre‐hospitalisation (PSQI) and in‐hospital sleep parameters (SF‐A) did not reveal significant differences in sleep duration (*p* = 0.85), sleep efficiency (*p* = 0.76) or nocturnal wake time (*p* = 0.89; Wilcoxon signed‐rank test). Overall, patients demonstrated notable sleep disturbances.

### 3.3 Actigraphy‐Based Observations

Actigraphy confirmed reduced activity between midnight and 8:00 AM compared with daytime (*p* = 0.0003), consistent with sleep periods. Mean activity‐related energy expenditure was 1103 ± 321 kcal/day; total daily expenditure 2.758 ± 487 kcal/day.

### 3.4 Postoperative ROS Concentrations in Patients With Different Cardiovascular Diseases

Across the 48‐h postoperative period, 13 systematically timed ROS measurements were obtained from each patient. Descriptively, ROS levels appeared to differ across surgical procedures (Table 1). CABG patients showed lower mean ROS values (5.2 ± 0.6 μM), whereas higher values were observed in more complex procedures, including the single LVAD implantation case (8.5 ± 2.6 μM). Given the very small subgroup sizes, particularly for procedures represented by only one patient, these findings are presented descriptively and should be interpreted with caution.

**TABLE 1:** Postoperative venous ROS concentrations (μM) according to type of cardiovascular surgery.

Surgical procedure	*n*	Minimum	Maximum	Mean ± SD (μM)	95% CI
CABG	8	3.9	6.2	5.2 **±** 0.6	4.9 to 5.6
AVR	2	3.6	7.4	5.8 **±** 1.1	5.1 to 6.5
LVAD	1	2.6	12.0	8.2	—
SVD repair	1	2.0	9.9	6.2	—
Thrombectomy	1	2.3	5.2	4.1	—
Aortic arch replacement	1	3.2	7.8	5.6	—
CRT	1	4.5	7.6	5.8	—

*Note:* Values represent mean ± SD of repeated measurements (13 samples per patient) obtained during the 48‐h postoperative period. For groups with *n* = 1, variability reflects within‐patient measurements and is therefore reported descriptively; standard deviations and confidence intervals are not applicable.

Abbreviations: μM, micromolar; AVR, aortic valve replacement; CABG, coronary artery bypass grafting; CRT, cardiac resynchronisation therapy; LVAD, Left Ventricular Assist Device; SVD, Sinus Venosus Defect.

### 3.5 ROS Associations With Baseline Characteristics

Female patients exhibited higher ROS levels than males (5.94 vs. 5.29 μM; unpaired *t*‐test: *t*(24) = 2.24, *p* = 0.0348).

No significant associations were found between ROS levels and BMI, age or time since surgery, though patients with adiposity grade I showed a nonsignificant trend toward higher ROS (normal weight: 5.2 ± 1.0 μM; overweight: 5.1 ± 0.7 μM; adiposity grade I: 6.0 ± 0.6 μM; *p* = ns).

### 3.6 Circadian Variations

Mean ROS appeared to vary across patients and over time within individual patients (Figure 1A). ROS values ranged from 0.9 to 14.7 μM, mean 5.6 ± 2.2 μM. No consistent circadian rhythm was evident; individual fluctuations ranged from −84.5% to +153.4% of the mean.

**FIGURE 1:**
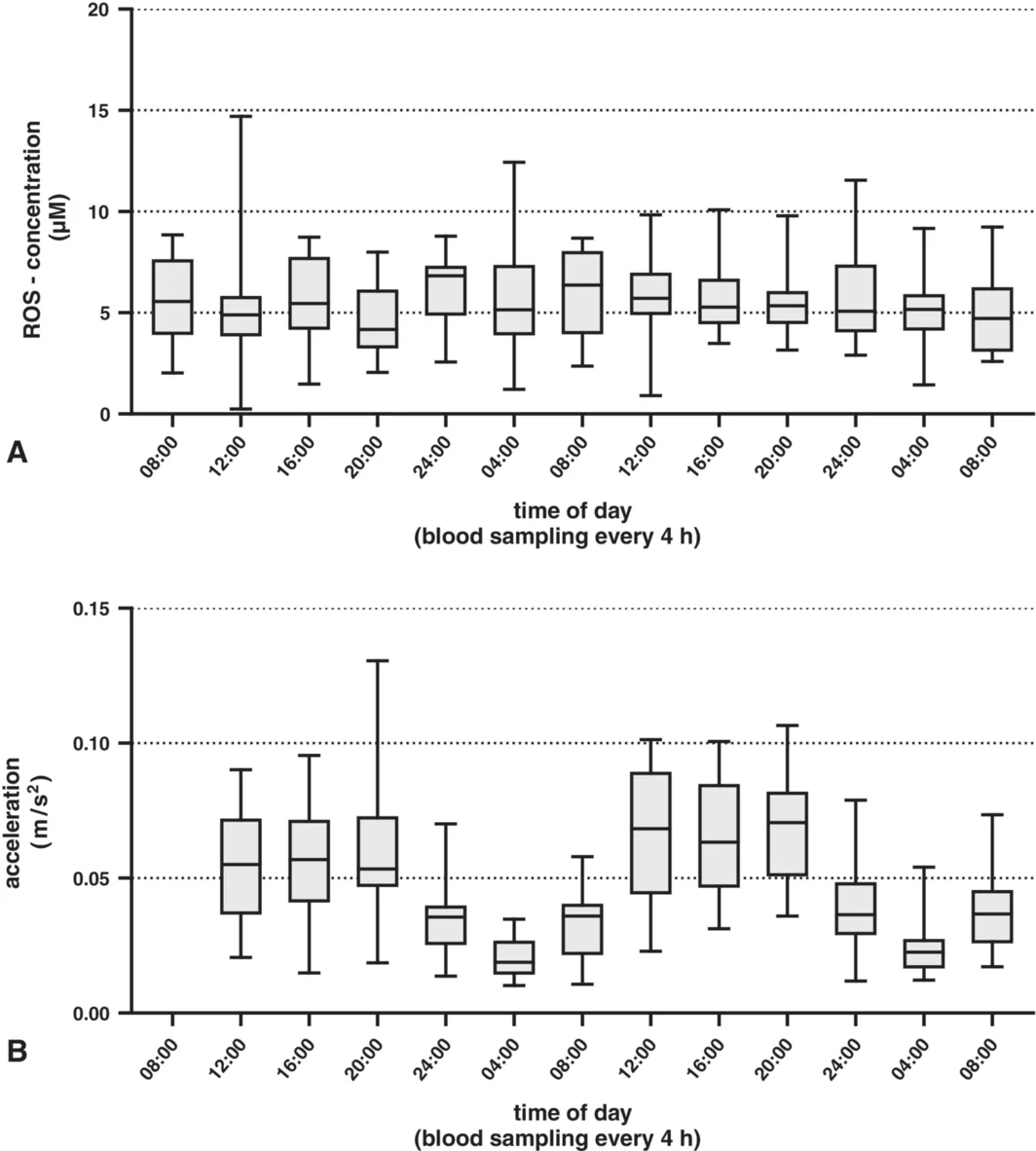
Boxplots of (A) the overall ROS concentrations (μM) over the diurnal cycle, and (B) wrist acceleration. The 8:00 acceleration value is missing since the monitor was attached at that time and recording began thereafter.

Exploratory 24‐h cosinor analyses using mixed‐effects models did not demonstrate statistically significant circadian rhythmicity at the group level (likelihood ratio test: *χ* ^2^(2) = 0.19, *p* = 0.91). At the individual level, no patient showed statistically significant rhythmicity (all *p* > 0.05). In contrast, the accelerometer measurements did reveal a clear diurnal pattern, with significantly reduced activity between 00:00 and 08:00 compared with the rest of the day (Figure 1B). Acceleration values ranged from 0.02 to 0.07, with a mean of 0.05 ± 0.02. Potential explanations include suppressed melatonin signalling, absence of external zeitgebers and confounding due to sedation or pain.

### 3.7 ROS and Sleep Correlations (Table 2)

**TABLE 2:** Correlations between subjective sleep parameters (ISI, PSQI and SF‐A) and mean ROS concentrations (M‐RC).

Variable	*r*	95% CI	*p*
Insomnia Severity Index (ISI)			
M‐RC vs. Severity	−0.15	−0.62 to 0.41	0.6
Pittsburgh Sleep Quality Index (PSQI)			
M‐RC vs. Global Score	0.40	−0.16 to 0.76	0.15
M‐RC vs. Sleep Latency	0.30	−0.26 to 0.71	0.27
M‐RC vs. Sleep Duration (h)	−0.49	−0.81 to 0.05	0.07+
M‐RC vs. Nocturnal Wake Time (min)^a^	0.62	0.16 to 0.86	0.01*
M‐RC vs. Sleep Efficiency	−0.74	−0.91 to −0.36	0.002**
M‐RC vs. Sleep Disturbance	−0.06	−0.56 to 0.48	0.85
M‐RC vs. Sleep Quality	0.19	−0.38 to 0.65	0.51
M‐RC vs. Daytime Dysfunction	0.21	−0.36 to 0.66	0.46
Sleep Questionnaire A (SF‐A)			
M‐RC vs. Sleep Duration (h)^a^	0.2	−0.35 to 0.65	0.47
M‐RC vs. Nocturnal Wake Time (min)^a^	−0.02	−0.53 to 0.50	0.94
M‐RC vs. Sleep Efficiency (%)	−0.04	−0.56 to 0.49	0.88
M‐RC vs. Sleep Quality	0.10	−0.45 to 0.59	0.72

Abbreviations: CI, confidence interval; ISI, Insomnia Severity Index; M‐RC, mean postoperative venous ROS concentration; PSQI, Pittsburgh Sleep Quality Index; *r*, correlation coefficient (Pearson or Spearman as appropriate); SF‐A, Sleep Questionnaire A.

^a^ Pearson correlation was applied where assumptions of normality were met; otherwise, Spearman rank correlation was used.

^+^ *p* ≤ 0.1.

* *p* ≤ 0.05.

** *p* ≤ 0.01.

Correlation analyses revealed no significant association between mean postoperative ROS concentrations and ISI scores, the PSQI global score or any SF‐A parameter. Among the PSQI subdomains, higher ROS concentrations were significantly associated with reduced sleep efficiency (*r* = −0.74, *p* = 0.002) and longer nocturnal wake time (*r* = 0.62, *p* = 0.01), while shorter sleep duration showed only a trend‐level association (*r* = −0.49, *p* = 0.07).

## 4 Discussion

This study suggests a link between postoperative sleep quality and oxidative stress. We demonstrate (1) feasibility of ROS quantification by ESR in a clinical setting; (2) substantial inter‐ and intraindividual variability in postoperative ROS levels; (3) absence of consistent circadian variation; and (4) associations between poor subjective sleep (reduced efficiency, shorter duration, more awakenings) and higher ROS.

Preoperative ROS levels vary according to the individual cardiovascular diseases, consistent with prior reports by our group (Beyersdorf 2024) and others (Sies et al. 2022; Sies et al. 2024; Cheng et al. 2022). In this study, we also observed intergroup variations in the immediate postoperative course (Table 1), and this supports our hypothesis that the duration, severity and extent of various cardiovascular diseases influence the pre‐ and postoperative status of the oxidative metabolism.

Female patients showed significantly higher ROS, echoing the conflicting literature (Kander et al. 2017; Brunelli et al. 2014) on sex differences in oxidative stress. Some studies propose oestrogen‐related protection (Kander et al. 2017), while others report higher oxidative stress in women (Brunelli et al. 2014). Future studies should test whether this association is reproducible in larger cohorts and investigate whether hormonal status, immune response or perioperative factors underlie sex‐specific differences in postoperative oxidative stress and related clinical outcomes.

Circadian rhythmicity of ROS, documented in controlled environments (Blanco et al. 2007), was not evident here (Figure 1B). Likely contributors include postoperative inflammation, hormonal dysregulation, sedation and environmental disruption (light, noise, interventions). Small sample size and interindividual variability may also mask rhythmicity.

The observed associations between specific sleep parameters and higher ROS concentrations support prior experimental work showing that sleep deprivation increases oxidative markers (Anafi et al. 2013; Vaccaro et al. 2020; Rorsman et al. 2025; Hill et al. 2018). Our exploratory findings suggest that selected aspects of subjective sleep continuity, particularly sleep efficiency and nocturnal wake time (Table 2), may be associated with postoperative oxidative stress. However, these associations were limited to specific questionnaire‐derived parameters and were not consistently supported by objective actigraphic measures. Human studies likewise report elevated oxidative stress in insomnia (Gulec et al. 2012) and shift workers (Teixeira et al. 2019). Our findings extend these observations to postoperative cardiac patients, suggesting that specific alterations in sleep architecture, rather than overall sleep quality, may influence redox balance during recovery. This is further supported by recent evidence indicating that sleep protects neuronal membranes against oxidative damage (Rorsman et al. 2025; Hill et al. 2018).

Individual variability in ROS profiles underscores the importance of personalised assessment.

Patients with high oxidative loads often had complex comorbidities or device support (e.g., LVAD), pointing to cumulative physiological burden. Oxidative stress in such patients may result from multiple sources, including systemic inflammation, mechanical shear forces, ischemia–reperfusion injury and immune activation (Mondal et al. 2013).

## 5 Strengths and Limitations

Strengths include combined biochemical, subjective and actigraphic data. Limitations are the small sample, lack of antioxidant marker measurement and absence of polysomnography for precise sleep staging.

A further limitation is the incomplete data acquisition in a subset of participants. Five patients did not complete the full protocol due to early removal of the central venous catheter or perceived study burden. This attrition may have introduced selection bias, as early catheter removal may be associated with a more favourable postoperative course, whereas participant burden may reflect reduced tolerance or clinical condition. Given the small sample size, this potential bias could not be formally assessed.

## 6 Future Perspectives

Larger multicentre studies should integrate circadian biomarkers (melatonin, cortisol), inflammatory cytokines and polysomnography. Interventions to improve postoperative sleep—behavioural, pharmacological or environmental—could potentially reduce oxidative stress and support recovery.

## 7 Conclusions

Postoperative cardiovascular patients show high variability in oxidative stress. While no statistically significant circadian rhythmicity was observed, specific sleep parameters—particularly reduced sleep efficiency and increased nocturnal wake time—were associated with elevated ROS concentrations. These findings suggest that targeted aspects of sleep may be linked to redox balance during postoperative recovery.

## Author Contributions

**K. Krawczyk:** investigation, writing – original draft, writing – review and editing, methodology, visualization, data curation, software. **T. Böhmerle:** investigation, formal analysis. **M. Czerny:** supervision. **J. Sahlmann:** supervision, validation, methodology. **D. Riemann:** conceptualization, investigation, writing – original draft, methodology, writing – review and editing, supervision, resources, data curation. **B. Feige:** investigation, methodology, formal analysis. **J. S. Pooth:** methodology, investigation, formal analysis. **A. Ayekoi:** data curation, investigation, methodology, validation. **E. Schleicher:** methodology, validation, writing – review and editing, supervision, resources, data curation. **F. Beyersdorf:** conceptualization, investigation, methodology, writing – original draft, writing – review and editing, data curation, resources, supervision, validation, visualization, project administration, formal analysis. **A. Günther:** investigation, methodology, data curation, validation.

## Funding

The authors have nothing to report.

## Conflicts of Interest

The authors declare no conflicts of interest.

## Data Availability

The data that support the findings of this study are available on request from the corresponding author. The data are not publicly available due to privacy or ethical restrictions.
